# 4.A. Workshop: Lessons from the response to COVID-19 to inform strengthening of Essential Public Health Functions

**DOI:** 10.1093/eurpub/ckac129.204

**Published:** 2022-10-25

**Authors:** Organised by: Health Information and Quality Authority, WHO, Department of Health in Ireland, Chair persons: Michelle O'Neill (Ireland), Ronan Glynn (Ireland)

## Abstract

Internationally, the COVID-19 pandemic has increased disease burden and mortality, impacted mental health and wellbeing and delayed diagnosis and treatment of non-COVID care. It has been argued that, had sufficient funding of Public Health, including pandemic preparedness, been in place historically, many shortcomings of the pandemic response could have been mitigated. Thus, strengthening Public Health systems is on the agenda of governments internationally. Areas of specific interest are, emergency preparedness, international cooperation and solidarity, promoting vaccination uptake, health equity and community engagement, health literacy and misinformation (particularly online), planning for future workforce requirements and harnessing digitalization in health to address communicable and non-communicable disease threats. The Essential Public Health Functions (EPHFs) provide a comprehensive, cost effective and operational approach to strengthening Public Health and are recognized by the WHO as key to building health system resilience. In a recent report, the WHO has proposed an approach to operationalizing the EPHFs, identifying key enablers that can be applied within specific country contexts. This workshop will describe the national and international research undertaken by the Irish Health Information and Quality Authority (HIQA) and the WHO at the request of the Department of Health to inform reform of the delivery model of Public Health in Ireland. HIQA and WHO will present the evidence base in terms of the value of EPHFs for health system strengthening, the findings of research to describe changes in Public Health structures and lessons learned in Ireland and across 12 countries during the COVID-19 pandemic, a description of the current state of delivery of the EPHFs within Ireland, and the results of a consultation survey distributed by the Department of Health in Ireland, investigating the experiences of organisations involved in the delivery of Public Health in light of the pandemic. The presenters will allow ample time for audience engagement and discussion with the expert panel to enable shared learning and to discuss the applicability of these findings to the reform and strengthening of the delivery of the EPHFs.

The workshop objectives are to:

• Describe the current evidence base in terms of the value of EPHFs for health system strengthening and the key enablers to support their application at country level.

• Describe the recent changes and lessons learned regarding the delivery of the EPHFs during the COVID-19 pandemic in 12 countries.

• Describe the delivery of the EPHFs in the Irish system in light of experience with COVID-19 and with awareness of current and future health system stressors.

• Describe Public Health organisations’ experiences of delivering the EPHFs in Ireland in light of the pandemic.

• Provide insights into how the delivery of the EPHFs could be reformed and strengthened for the future.

**Key messages:**

• Lessons from the Public Health response to COVID-19, internationally and in Ireland will be discussed.

• The workshop will provide a space to share ideas on reform and strengthening EPHF delivery.

